# Pigment Epithelium-Derived Factor (PEDF) mediates cartilage matrix loss in an age-dependent manner under inflammatory conditions

**DOI:** 10.1186/s12891-017-1410-y

**Published:** 2017-01-25

**Authors:** Daisy S. Nakamura, Judith M. Hollander, Tomoya Uchimura, Heber C. Nielsen, Li Zeng

**Affiliations:** 1 Program in Cell, Molecular and Developmental Biology, Sackler School of Graduate Biomedical Sciences, Tufts University, Boston, MA USA; 2 Department of Pediatrics, Tufts Medical Center, Boston, MA USA; 3 Department of Integrative Physiology and Pathobiology, Tufts University School of Medicine, Boston, MA USA; 4 Department of Orthopaedics, Tufts Medical Center, Boston, MA USA

**Keywords:** Pigment epithelium-derived factor, Matrix, cartilage, IL-1β, MMPs, Arthritis, Inflammation, SERPINF1, Age-related

## Abstract

**Background:**

Inflammation is a major cause of cartilage destruction and leads to the imbalance of metabolic activities in the arthritic joint. Pigment epithelium-derived factor (PEDF) has been reported to have both pro- and anti-inflammatory activities in various cell types and to be upregulated in the arthritic joint, but its role in joint destruction is unclear. Our aim was to investigate the role of PEDF in cartilage degeneration under inflammatory conditions.

**Methods:**

PEDF was ectopically expressed in primary human articular chondrocytes, and catabolic gene expression and protein secretion in response to the pro-inflammatory cytokine interleukin 1 beta (IL-1β) were evaluated. Metatarsal bones from PEDF-deficient and wild type mice were cultured in the presence or absence of IL-1β. Cartilage matrix integrity and matrix metalloproteinases MMP-1, MMP-3, and MMP-13 were evaluated. PEDF-deficient and wild type mice were evaluated in the monosodium iodoacetate (MIA) inflammatory joint destruction animal model to determine the role of PEDF in inflammatory arthritis in vivo. Student’s *t*-tests and Mann–Whitney tests were employed where appropriate, for parametric and non-parametric data, respectively.

**Results:**

We showed that PEDF protein levels were higher in human osteoarthritis samples compared to normal samples. We demonstrated that ectopic PEDF expression in primary human articular chondrocytes exacerbated catabolic gene expression in the presence of IL-1β. In whole bone organ cultures, IL-1β induced MMP-1, MMP-3 and MMP-13 protein production, and caused significant cartilage matrix loss. Interestingly, Toluidine Blue staining showed that PEDF-deficient bones from 29 week old animals, but not 10 week old animals, had reduced matrix loss in response to IL-1β compared to their wild type counterparts. In addition, PEDF-deficiency in 29 week old animals preserved matrix integrity and protected against cell loss in the MIA joint destruction model in vivo.

**Conclusion:**

We conclude that PEDF exacerbates cartilage degeneration in an age-dependent manner under an inflammatory setting. This is the first study identifying a specific role for PEDF in joint inflammation and highlights the multi-faceted activities of PEDF.

**Electronic supplementary material:**

The online version of this article (doi:10.1186/s12891-017-1410-y) contains supplementary material, which is available to authorized users.

## Background

Arthritis afflicts 52.5 million Americans [1] and is a highly prevalent disease worldwide [2]. It presents clinically as joint pain and immobility, affecting multiple joints (e.g. hands, feet, knee, hips) and joint tissues (e.g. cartilage, synovium, subchondral bone). While juveniles also suffer from arthritis, the risk of developing arthritis increases with age. Other major risk factors include obesity, joint injury and family history. A key feature of arthritis is inflammation, where pro-inflammatory cytokines such as interleukin 1 beta (IL-1β) are important mediators. In fact, in mouse models of osteoarthritis (OA) and rheumatoid arthritis (RA), inhibiting IL-1 activity inhibits arthritis progression [3–6].

IL-1β stimulates chondrocytes to release various matrix metalloproteinases (MMPs), including MMP-1, MMP-3, and MMP-13 [7–10], which are key regulators of matrix destruction. In fact, cartilage erosion in a surgical model of OA is dependent on MMP-13 activity [11] and local IL-1 receptor antagonist (IL-1Ra) therapy reduces degenerative changes in the joint in a traumatic injury-induced OA mouse model [6]. However, monotherapy clinical trials with IL-1Ra or monoclonal antibodies targeting the IL-1 receptor have resulted in variable results for OA [12, 13]. For RA, clinical trials with the IL-1Ra were beneficial only for a subset of the highest dose cohort [14]. This suggests that additional modulators may collaborate with IL-1β to drive inflammation-associated joint destruction, highlighting the complexity and incomplete understanding of arthritis pathogenesis.

One such modulator may be pigment epithelium-derived factor (PEDF), which is a 50kD secreted glycoprotein belonging to the non-inhibitory family of serine protease inhibitors (Serpins) [15, 16]. It is encoded by the SERPINF1 gene and expressed in multiple tissues [17–19]. PEDF has been extensively studied for its anti-angiogenic and neurotrophic properties [18, 20–25]. It has also been shown to modulate the inflammatory response in a variety of systems. For example, systemic expression of PEDF in diabetic rats led to a reduction in tumor necrosis factor alpha (TNFα) and monocyte chemoattractant protein 1 (MCP-1) expression in the retina as well as the kidney [26, 27]. On the other hand, in skeletal muscle cells and neonatal astrocytes, PEDF treatment activated the inflammatory mediator, nuclear factor of kappa light polypeptide gene enhancer in B-cells (NF-κB) [28, 29].

In arthritic disease pathogenesis, the role of PEDF is unclear as even its expression in the joint is controversial. In one report, PEDF expression is found in normal articular chondrocytes and upregulated in human OA cartilage samples [30]. Another recent study shows that PEDF is not expressed in normal or OA articular chondrocytes, but is only upregulated predominantly in osteophytic chondrocytes [31]. While it was shown that PEDF contributes to the terminal differentiation of the endochondral ossification process of osteophyte formation [31], its role in the inflammatory process in joint destruction remains unclear. In this study, we sought to investigate PEDF protein in articular chondrocytes, and address whether PEDF plays a role in cartilage degeneration by evaluating its effects on cartilage in an ex vivo culture system under inflammatory stimuli and in an inflammatory animal model of joint destruction.

## Methods

### Normal and OA Human Cartilage Specimens

Normal human articular cartilage slices were isolated from the tibial plateau of cadaveric joints obtained from the National Disease Research Interchange. Human OA articular cartilage slices were isolated from the tibial plateau of resected knees from patients undergoing total knee replacement surgery at Tufts Medical Center (age/sex: 54/F, 67/M, 53/F), and cartilage damage from these samples were confirmed through histological analysis [32].

### Generating Lentiviral Constructs

The plasmid containing the human SERPINF1 gene was a generous gift from Dr. S. Patricia Becerra (NIH). The human SERPINF1 gene containing signal sequences and a tdTomato sequence were amplified with P2A-sequence-containing primers. Overlapping sequences of human SERPINF1-P2A and P2A-tdTomato were used in Gibson Assembly with the pWPXLd lentiviral backbone (Addgene), targeting SERPINF1-P2A-tdTomato downstream of the EF-1α promoter. Similarly, the GFP sequence was amplified with P2A-sequence-containing primers. Overlapping GFP-P2A and P2A-tdTomato sequences were used in Gibson Assembly with the pWPXLd lentiviral backbone for use as a transduction control.

### Cell Culture and Reverse Transcriptase-Polymerase Chain Reaction (RT-PCR) Analysis

Primary normal human articular chondrocytes (Lonza) were redifferentiated in alginate beads per the manufacturer's protocol using chondrogenic differentiation medium (Lonza). Redifferentiated normal human articular chondrocytes were transduced with lentiviruses expressing GFP or PEDF for 48 h, then cultured for 4 days in chondrogenic differentiation medium in the presence or absence of 1 ng/mL of interleukin-1β (IL-1β) (PeproTech). Total RNA from cultured chondrocytes was isolated using the RNeasy Mini Kit (Qiagen) and complementary DNA was generated using Moloney Murine Leukemia Virus Reverse Transcriptase (Invitrogen). Quantitative PCR was performed using an iQ5 Real-Time PCR Detection System (Bio-Rad). All RT-PCR analyses were normalized to TATA box binding protein mRNA expression [33]. Primer sequences are available from the author upon request.

### Western blot

Redifferentiated primary normal human articular chondrocytes (Lonza) were transduced with lentiviruses expressing GFP or PEDF for 72 h. Primary normal human articular chondrocytes were lysed using the standard RIPA buffer. Nuclear and cytoplasmic fractions were obtained using NE-PER Nuclear and Cytoplasmic Extraction Reagents (Pierce) per manufacturer instructions. Protein concentrations were determined using the DC Protein Assay (Bio-Rad). Cytoplasmic and nuclear fraction proteins (5 μg) were loaded onto gels. Primary and secondary antibodies: rabbit anti-PEDF (BioProductsMD, AB-PEDF), mouse anti-α-tubulin (Developmental Studies Hybridoma Bank, 12G10), mouse anti-TATA binding protein (TBP) (Thermo Scientific, 51841), and goat anti-rabbit or mouse IgG, (H + L) HRP conjugate (Millipore/Chemicon).

### ELISA

PEDF, MMP-1, MMP-3, and MMP-13 protein levels were determined by ELISA using the following kits from RayBiotech: PEDF (ELH-SerpinF1-1), MMP-1 (ELH-MMP1-1), MMP-3 (ELH-MMP3-1), and MMP-13 (ELH-MMP13-1). Conditioned media was collected from primary normal human articular chondrocytes transduced with a lentiviral GFP or lentiviral PEDF for four days in the presence or absence of 1 ng/mL of IL-1β. ELISA was performed per manufacturer instructions.

### Animals and MIA Injection

All animal care and experimental procedures were approved by the Institutional Animal Care and Use Committee at Tufts University. Serpinf1−/− mice were a generous gift from Regeneron to Dr. Nielsen. These mice have been previously described to lack PEDF protein production [19, 34–38], thus the Serpinf1−/− mice are referred to as PEDF-deficient mice hereafter. Mice were housed in groups under standard conditions with a 12 h light/dark cycle and allowed *ad libitum* access to standard chow and water. 29 week old mice were anesthetized by isoflurane/O_2_ inhalation and received a single intraarticular injection of monosodium iodoacetate (MIA) (Sigma) using a 30G ½” needle with 50 μg MIA dissolved in 5 μL of sterile PBS. PBS-injected contralateral knees served as negative controls. Knees were harvested 10 days post-injection.

### Metatarsal bone culture

Metatarsal bones from 10 or 29 week old wild type or PEDF-deficient mice were harvested and the second, third and fourth metatarsal bones were cultured in the presence or absence of 10 ng/mL IL-1β for 7 days in Dulbecco's Modified Eagle Medium (Invitrogen) supplemented with 0.25% Fetal Bovine Serum, 0.28% Ascorbic Acid, 0.25% Sodium Pyruvate and 1% Antibiotic-Antimycotic (Invitrogen). Media were replaced every 48 h.

### Immunohistochemistry (IHC)

For the evaluation of human articular cartilage, sections were fixed in 1% paraformaldehyde (PFA) in PBS overnight at 4 °C, decalcified in 0.33 M EDTA, embedded in paraffin and sectioned at 5 μm thickness. For cartilage matrix staining, human cartilage sections were stained with 0.1% Safranin O and counterstained with Fast Green and Hematoxylin. For PEDF staining, sections were treated sequentially with both heat-mediated and enzymatic antigen retrieval using 10 mM Sodium Citrate, pH 6.0 and 0.3% hyaluronidase supplemented with 0.15% trypsin-EDTA, respectively. Samples were blocked in 10% goat serum in PBS, followed by mouse anti-PEDF (Chemicon, 10F12.2) incubation overnight, and biotinylated anti-mouse IgG (Vector Laboratories) incubation for 2 h. Primary antibody staining was visualized using the Vectastain ABC Kit with DAB Peroxidase Substrate Kit (Vector Laboratories). Samples were counterstained with 0.5% Methyl Green in 0.1 M Sodium Acetate.

For the evaluation of mouse knees, isolated joints were fixed in 1% PFA in PBS overnight at 4 °C, decalcified in 0.33 M EDTA, embedded in paraffin and serial sagittal sections of 5 μm thickness were obtained. For the evaluation of metatarsal bones, samples were fixed and decalcified as the knee joints, embedded in O.C.T. (Tissue-Tek) and cryosectioned at 5 μm thickness. For histological analysis, sections were stained with 0.4% Toluidine Blue, or with 0.1% Safranin O and counterstained with Fast Green and Hematoxylin. For immunostaining, all mouse sections were sequentially treated with both heat-mediated and enzymatic antigen retrieval, as in the human samples. The primary antibodies were: mouse anti-PEDF (Chemicon, 10F12.2), rabbit anti-MMP-1 (Proteintech, 10371-2-AP), rabbit anti-MMP-3 (Proteintech, 17873-1-AP) and mouse anti-MMP-13 (Abcam, VIIIA2). For PEDF fluorescence staining, Alexa Fluor 594 Goat anti-mouse secondary antibody (Jackson ImmunoResearch Laboratories) was used. For MMP-13 fluorescence staining, biotinylated horse anti-mouse IgG (Vector Laboratories) and DyLight 594 Streptavidin (Vector Laboratories) were used. Nuclei were stained with DAPI (Roche) in both cases. For colorimetric staining, a biotinylated anti-mouse IgG secondary antibody (Vector Laboratories) or a goat anti-rabbit IgG secondary antibody (Vector Laboratories) was used, followed by the Vectastain ABC Kit with DAB Peroxidase Substrate Kit (Vector Laboratories), and counterstained with 0.5% Methyl Green in 0.1 M Sodium Acetate. For PEDF and MMP-13 IHC, which involved the use of mouse antibodies, sections were blocked with M.O.M. Mouse IgG Blocking Reagent (Vector Laboratories) to reduce non-specific binding. For MMP-1 staining, endogenous biotin was blocked with an Avidin/Biotin Blocking Kit (Vector Laboratories) before incubation with the anti-MMP-1 antibody, and endogenous peroxidase activity was quenched by 3% hydrogen peroxide after incubation with the primary antibody.

Bright-field and fluorescent images were taken using an Olympus IX-71 inverted microscope and an Olympus DP70 or DP80 digital camera.

### Histological analysis

Image analysis was performed using the ImageJ software. For percent cartilage matrix loss along the articular surface, the length of regions exhibiting a loss of Toluidine Blue staining along the articular surface were measured. The sum of these lengths was divided over the total articular surface length to obtain a percent surface area loss. For percent positive area, regions of interest were drawn around the articular cartilage and percent positive areas of MMP-13 staining were calculated. Using the Osteoarthritis Research Society International (OARSI) histological scoring system, OARSI scores were tallied according to the modified scoring system previously described for osteoarthritis models with limited structural damage [39]. Percent cell loss was calculated by comparing DAPI stained images to bright-field images [40]. Specifically, the total cell number was calculated as the total number of lacunae within the articular cartilage. Percent cell loss is the ratio of the number of empty lacunae (i.e. DAPI-negative) over total lacunae in the articular cartilage.

### Statistical analysis

Data were plotted as mean ± SEM. Parametric (Student’s *t*-test) and non-parametric (Mann–Whitney test) statistical analyses were employed where appropriate. All statistical analyses were performed using Prism (GraphPad Software). *P*-values of < 0.05 were considered statistically significant.

## Results

### PEDF overexpression potentiates the catabolic effect of IL-1β in primary human articular chondrocytes

**Fig. 1:**
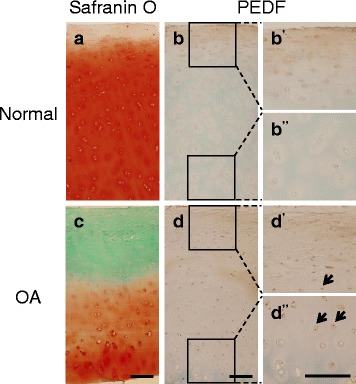
PEDF expression is elevated in human osteoarthritis (OA) cartilage specimens. Normal samples were obtained from the National Disease Research Interchange and OA samples were obtained from patients undergoing total knee replacement surgery. **a** Normal sample stained with Safranin O and counterstained with Hematoxylin and Fast Green. Mankin score = 1. **b** Immunohistochemistry (IHC) analysis on a normal cartilage sample using a mouse anti-PEDF antibody and counterstained with Methyl Green. Magnified superficial (b') and deeper (b'') zones are shown from insets. **c** OA sample stained with Safranin O and counterstained with Hematoxylin and Fast Green. Mankin score = 5. **d** IHC analysis on an OA sample using a mouse anti-PEDF antibody and counterstained with Methyl Green. Magnified superficial (d') and deeper (d'') zones are shown from insets. Arrows indicate positive PEDF staining. A negative control using a biotinylated horse anti-mouse IgG secondary antibody alone is shown in the Additional file 2: Figure S1. Scale bar = 200 μm

Because of the controversy surrounding PEDF levels in chondrocytes, we first performed immunohistochemistry to assess PEDF protein location and levels. We obtained articular cartilage samples from cadaveric knees of normal healthy subjects and osteoarthritis (OA) samples from patients undergoing total knee replacement surgery. As expected, OA knees exhibited greater cartilage matrix loss, as indicated by reduced Safranin O staining, and scored higher on the Mankin scale, which classifies samples according to OA histopathology [32] (Fig. 1a, c). Samples with greater damage demonstrated elevated PEDF protein levels compared to normal control samples (Fig. 1b, d), supporting the notion that PEDF is indeed present in articular chondrocytes and elevated in OA samples [30].

**Fig. 2:**
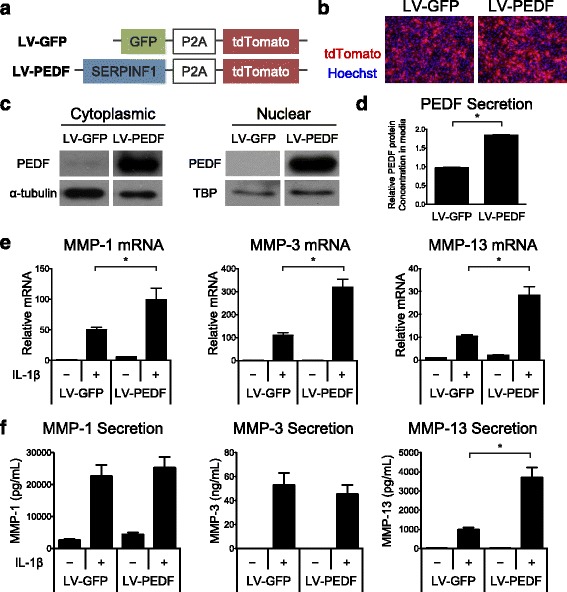
PEDF ectopic expression upregulates catabolic gene expression in normal human articular chondrocytes (NHACs). **a** Lentiviral constructs were generated that contained the human SERPINF1 gene, which encodes for the PEDF protein, and a tdTomato moiety that was separated from the SERPINF1 gene by a P2A self-cleaving peptide (LV-PEDF). A lentiviral construct encoding the gene for green fluorescent protein (GFP) and a tdTomato moiety (LV-GFP) was also constructed. **b** tdTomato expression is comparable between the two expression vectors. **c** Western Blot analysis confirmed elevated PEDF protein production in the cytoplasm and nucleus of NHACs transduced with LV-PEDF. α-tubulin was used as a loading control for cytoplasmic proteins. TATA binding protein (TBP) was used as a loading control for nuclear proteins. LV-GFP: GFP-encoding lentivirus; LV-PEDF: PEDF-encoding lentivirus. **d** ELISA analysis of PEDF protein secretion into the media of NHACs transduced with LV-GFP or LV-PEDF following 4 days in culture. Data are represented as fold change relative to LV-GFP samples. **e** Quantitative RT-PCR analysis of MMP-1, MMP-3 and MMP-13 on NHACs transduced with LV-GFP or LV-PEDF and cultured in the presence or absence of 1 ng/mL IL-1β for 4 days. Data are represented as fold change relative to LV-GFP-transduced cells in the absence of IL-1β within each gene. Gene expression was normalized to TATA-binding protein mRNA expression levels. **f** ELISA analysis of MMP-1, MMP-3 and MMP-13 protein secreted into the media of NHACs transduced with LV-GFP or LV-PEDF and cultured in the presence or absence of 1 ng/mL IL-1β for 4 days. Data are presented as fold change relative to that found in the media of LV-GFP-transduced cells cultured in the absence of IL-1β. All data are plotted as mean ± SEM. * *p* < 0.05 (Mann–Whitney test)

To study the effects of elevated PEDF levels on chondrocytes, we generated a lentiviral construct that contained the human SERPINF1 gene, which encodes for the PEDF protein, and a tdTomato moiety that was separated from the SERPINF1 gene by a P2A self-cleaving peptide. This construct was termed lentivirus PEDF (LV-PEDF). A lentiviral construct encoding the gene for green fluorescent protein (GFP) (LV-GFP) was also constructed and served as a transduction control (Fig. 2a). We obtained comparable lentiviral expression for human PEDF and control GFP after transducing the expression vectors into normal human articular chondrocytes (NHACs) (Fig. 2b). Western Blot analysis showed that ectopically expressed PEDF protein was detected in the cytoplasm as well as in the nucleus (Fig. 2c). In addition, ELISA analysis indicated that PEDF was secreted into the culture media (Fig. 2d). These findings are consistent with prior reports showing differential PEDF localization [15, 41–46]. Next, we investigated the role of PEDF in chondrocytes under inflammatory conditions by transducing NHACs with LV-PEDF followed by treating with 1 ng/mL of the pro-inflammatory cytokine, interleukin-1β (IL-1β), for 4 days. In the absence of IL-1β, PEDF overexpression alone slightly elevated the mRNA expression of catabolic genes (*MMP-1*, *MMP-3,* and *MMP-13*) relative to GFP-transduced control NHACs. While IL-1β treatment significantly elevated catabolic gene expression in the GFP-transduced control cells, additional ectopic PEDF expression synergized with IL-1β, leading to further elevation of catabolic gene expression (Fig. 2e). To examine the effect of PEDF ectopic expression on MMP secretion, we performed ELISA on the culture media of NHACs transduced with LV-GFP or LV-PEDF. IL-1β treatment significantly elevated MMP-1, MMP-3, and MMP-13 secretion (Fig. 2f). Lentiviral PEDF overexpression did not further augment MMP-1 and MMP-3 secretion, but strongly enhanced MMP-13 secretion (Fig. 2f). It would be interesting to determine whether PEDF enhances MMP-1 and MMP-3 secretion from chondrocytes under longer culture times, or if PEDF altered IL-1β-induced MMP activities over time. Nevertheless, these results suggest that PEDF potentiates the catabolic effects of IL-1β.

### PEDF is required to potentiate IL-1β-induced cartilage matrix loss in articular cartilage

To examine whether PEDF played a similar role in chondrocytes embedded in their native cartilage environment, we sought to evaluate matrix levels in metatarsal bones from wild type and PEDF-deficient mice challenged with inflammatory stimuli. Because age is a major risk factor for arthritis, we evaluated mouse metatarsal bones from two different ages, one of early adulthood (10 weeks old) in the mouse and one of later adulthood (29 weeks old). We opted to culture metatarsal bones for their ease of organ culture and susceptibility to inflammation-mediated arthritic damage in culture [47–50].

**Fig. 3:**
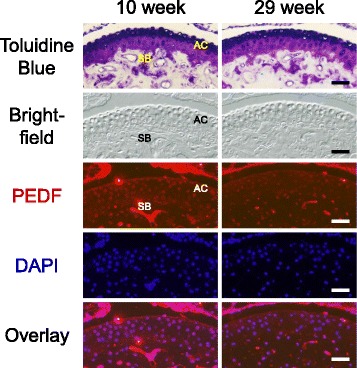
Detection of PEDF in the articular surface of adult mouse metatarsal bones. Metatarsal bones from 10 and 29 week old mice were subjected to histological analysis by Toluidine Blue staining. AC: articular cartilage. SB: subchondral bone. Immunohistochemistry (IHC) analysis of PEDF protein was performed on sections adjacent to those used for Toluidine Blue staining. Nuclei were stained with DAPI. Bright field, PEDF staining (red), DAPI and PEDF/DAPI overlay images of the same section are shown. A negative control using goat anti-mouse IgG secondary antibody alone is shown in the Additional file 2: Figure S1. Scale bar = 200 μm

**Fig. 4:**
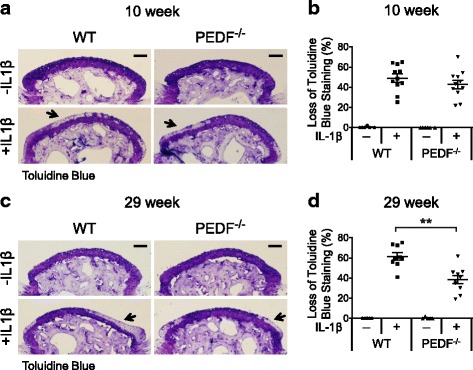
PEDF-deficiency protects against IL-1β-induced matrix loss in organ cultures of metatarsal bones. **a** Metatarsal bones from 10 week old wild type or PEDF-deficient mice were harvested and cultured in the presence or absence of 10 ng/mL IL-1β for 7 days. Samples were cryosectioned and stained with Toluidine Blue. Scale bar = 100 μm. **b** Percent loss of Toluidine Blue staining along the articular surface in 10 week old samples with respect to the total surface area was calculated. **c** Metatarsal bones from 29 week old wild type or PEDF-deficient mice were harvested and cultured in the presence or absence of 10 ng/mL IL-1β for 7 days. Samples were cryosectioned and stained with Toluidine Blue. Scale bar = 100 μm. **d** Percent loss of Toluidine Blue staining along the articular surface in 29 week old samples with respect to the total surface area was calculated. For experiments involving either 10 or 29 week old mice, the middle three metatarsal bones isolated from three animals were used. Each data point in the graphs represents an individual metatarsal bone. Each treatment was repeated 5–11 times. Data are plotted as mean ± SEM. ** *p* = 0.0021 (Mann–Whitney test)

We first confirmed that PEDF protein was found in the articular chondrocytes of metatarsal bones of both 10 and 29 week old mice (Fig. 3). Because PEDF secretion and extracellular matrix (ECM) binding of PEDF are both well-established [15, 42, 43, 46, 51–53], it is conceivable that we detected PEDF in both extracellular and intracellular compartments. We next performed Toluidine Blue staining to evaluate cartilage matrix levels. We utilized the constitutive PEDF-deficient mouse that lacks the Serpinf1 gene which encodes PEDF [19, 34–38]. We found no difference in cartilage matrix levels between wild type and PEDF-deficient explant cultures in the absence of IL-1β treatment. However, in the presence of IL-1β, samples from both 10 and 29 week old mice demonstrated a loss of Toluidine Blue staining along the articular surface (Fig. 4a, c). Although a more centrally-located matrix loss has been found in osteoarthritic joints [11, 39, 54], matrix loss in these bones after IL-1β treatment was present across the entire length of the articular surface, possibly a result of absent mechanical stress in the ex vivo culture condition in contrast to the in vivo setting. Quantification of percent surface area loss of staining upon IL-1β treatment showed no statistically significant difference between PEDF-deficient mice and their wild type counterparts in samples isolated from 10 week old mice (Fig. 4b). Strikingly, we observed significantly lower levels of cartilage matrix loss in the PEDF-deficient samples isolated from 29 week old mice compared to their wild type counterparts, suggesting that PEDF-deficiency attenuated this loss of surface matrix in samples from older mice under inflammatory conditions (Fig. 4d). Additionally, cartilage matrix loss was significantly greater in wild type samples isolated from 29 week old mice (mean of 61%) compared to 10 week old mice (mean of 49%), supporting the notion that older animals are more susceptible to damage [54] (Fig. 4b, d).

**Fig. 5:**
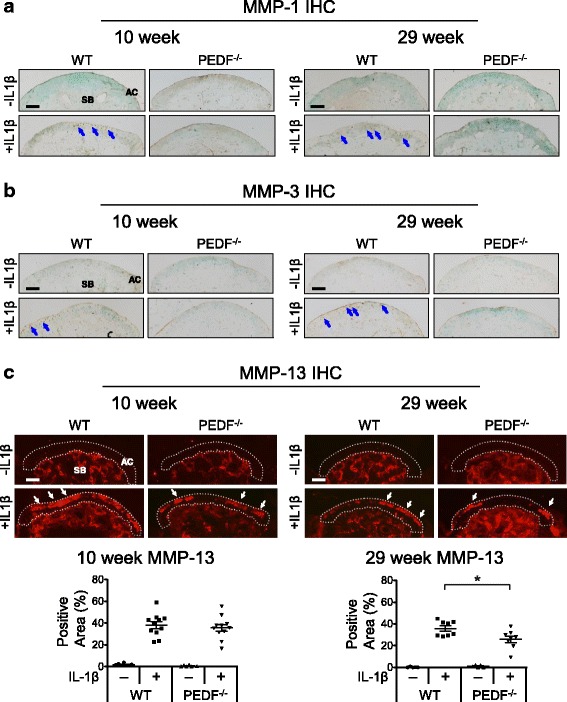
PEDF-deficiency protects against IL-1β-induced MMP-1, MMP-3 and MMP-13 protein production in metatarsal bone cultures. Metatarsal bones from 10 and 29 week old wild type or PEDF-deficient mice were harvested and cultured in the presence or absence of 10 ng/mL IL-1β for 7 days. Cryosectioned samples were analyzed for MMP protein levels by immunohistochemistry (IHC). AC: articular cartilage. SB: subchondral bone. Scale bar = 100 μm. **a** Samples were stained with rabbit anti-MMP-1 and counterstained with 0.5% Methyl Green in 0.1 M Sodium Acetate. Arrows indicate positive MMP-1 staining. **b** Samples were stained with rabbit anti-MMP-3 and counterstained with 0.5% Methyl Green in 0.1 M Sodium Acetate. Arrows indicate positive MMP-3 staining. **c** Samples were stained with mouse anti-MMP-13 and nuclei were stained with DAPI. Arrows indicate positive MMP-13 staining. The percent area staining positive for MMP-13 in the 10 and 29 week old samples was calculated as the area with MMP-13 staining along the articular cartilage area against the total articular cartilage area. For experiments involving either 10 or 29 week old mice, the middle three metatarsal bones isolated from three animals were used. Each data point in the graphs represents an individual metatarsal bone. Each treatment was repeated 5–11 times. Negative controls using goat anti-rabbit IgG or biotinylated horse anti-mouse IgG followed by streptavidin DyLight 594 alone are shown in the Additional file 2 Figure S1. Data are plotted as mean ± SEM. * *p* = 0.0281 (Mann–Whitney test)

We next assessed MMP protein levels within the articular cartilage. Neither wild type nor PEDF-deficient samples expressed significant amounts of MMP-1, MMP-3, or MMP-13 within the articular cartilage in the absence of IL-1β. However, in the presence of an inflammatory stimulus, their levels were induced (Fig. 5a-c). Specifically, articular cartilage from 10 week old PEDF-deficient mice expressed MMP-13 levels similar to their age-matched wild type controls under IL-1β treatment, while articular cartilage from 29 week PEDF-deficient mice expressed lower levels of MMP-13 compared to their wild type counterparts (Fig. 5c). We observed a similar trend in MMP-1 and MMP-3 protein levels in the PEDF-deficient samples (Fig. 5a-b). However, as images of colorimetric histology are not reliable for quantitative analysis, we did not attempt to quantify the amount of MMP-1 and MMP-3 proteins in these samples.

### PEDF potentiates joint cartilage damage in an inflammatory joint destruction model in vivo

We next evaluated the role of PEDF in regulating cartilage integrity in vivo using the monosodium iodoacetate (MIA) model. MIA is a specific inhibitor of glycolysis which causes cell death and induces joint inflammation and cartilage damage [40]. Because we observed a protective effect of PEDF-deficiency in the older animals (29 weeks old) in our organ cultures, we evaluated the role of PEDF in vivo using animals of this age.

**Fig. 6:**
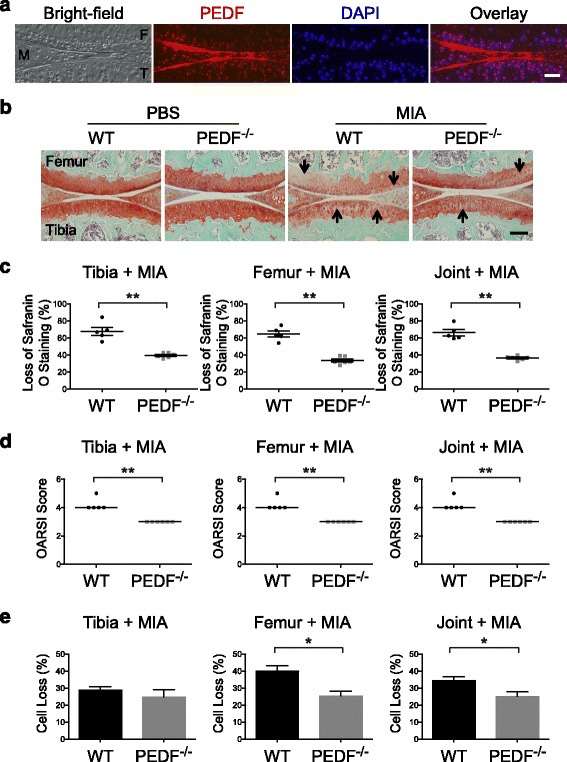
PEDF-deficiency protects against monosodium iodoacetate (MIA)-induced joint cartilage damage. **a** Immunofluorescence staining was used to determine PEDF protein levels in knee joints from 29 week old wild type animals using a mouse anti-PEDF antibody. Nuclei were stained with DAPI. A negative control using goat anti-mouse IgG secondary antibody alone is shown in the Additional file 2: Figure S1. M: meniscus; F: femur; T: tibia. Scale bar = 200 μm. **b** MIA or PBS was intraarticularly injected into 29 week old wild type or PEDF-deficient knees, which were harvested after 10 days. Samples were stained with Safranin O and counterstained with Hematoxylin and Fast Green. Arrows indicate areas exhibiting a loss of Safranin O staining. Scale bar = 100 μm. **c** The percent area loss of Safranin O staining along the articular surface was calculated for the femur and tibia independently, as well as for the whole joint (femur + tibia). Each point represents the averaged score of an individual animal. Data are plotted as mean ± SEM. **d** OARSI scores were calculated from Safranin O stained joints. Each point represents an individual animal. The bar represents the median. **e** Quantification of percent cell loss based on DAPI cell counts of the articular surface and empty lacunae. ** *p* < 0.01 (Mann–Whitney test) * *p* < 0.05 (Student’s *t*-test)

We first confirmed that PEDF protein was present in the articular chondrocytes of the mouse knee joint (Fig. 6a). Safranin O staining showed comparable matrix levels between wild type and PEDF-deficient animals in the vehicle control groups, suggesting that the loss of PEDF alone did not affect the formation of articular cartilage. In contrast, cartilage matrix was reduced in MIA-injected wild type knees, but was better preserved in the MIA-injected PEDF-deficient knee (Fig. 6b). When the percent loss of Safranin O staining within the articular cartilage was quantified for MIA-injected knees, the tibia and femur from PEDF-deficient mice were better protected from matrix loss and resulted in better preserved matrix within the joint (Fig 6c). Using the Osteoarthritis Research Society International (OARSI) histological scoring system [39], our measurements of percent loss of Safranin O staining corresponded to OARSI scores that also reflected less damage with PEDF-deficiency (Fig. 6d).

We also evaluated the percentage of cell loss based on the previously published analysis method that compared empty lacunae and DAPI staining [40]. While the total cell numbers between wild type and PEDF-deficient knees were not significantly different (See Additional file 1), cell loss was widespread in MIA-treated joints [55–57]. We found a substantial decrease in percent cell loss in PEDF-deficient knees relative to their wild type controls (Fig. 6e). Interestingly, the tibia did not show a significant reduction of cell loss in the PEDF-deficient animals, despite a significant reduction of matrix loss (Fig. 6c). Taken together, these data suggest that PEDF exacerbates cartilage damage in an inflammatory environment and that its presence over time enhances the competence of chondrocytes to respond to inflammatory stimuli.

## Discussion

In this study, we demonstrated that PEDF protein is expressed in adult knee articular chondrocytes where it potentiates inflammatory stimuli-induced joint cartilage damage. PEDF enhanced the catabolic profile of primary human articular chondrocytes under inflammatory stimuli. In explant cultures using PEDF-deficient mice, loss of PEDF expression protected the explants cultured from older mice against matrix loss. The protective effect was associated with a decrease in MMP protein levels. In an in vivo inflammatory joint destruction model, PEDF loss preserved matrix levels and cellularity. These data are consistent with the notion that PEDF renders chondrocytes more responsive to inflammatory stimuli over time.

Prior to this work, there were conflicting reports regarding the expression of PEDF in the diseased joint, despite reports of its expression during cartilage development in proliferating and pre-hypertrophic chondrocytes within the growth plate [17]. One report showed that PEDF is upregulated in osteophytes [31], the formation of which involves chondrocyte hypertrophy and osteoblast formation. This is consistent with other reports that indicate PEDF, under normal physiological conditions, promotes osteoblast and osteoclast differentiation and bone mineralization [58–60]. Our study supports the work of Pfander et al., indicating that PEDF is indeed expressed in articular cartilage and is upregulated under osteoarthritic conditions.

The role of PEDF under inflammatory conditions in cartilage or bone has not been previously explored. Our in vitro and in vivo studies indicate that PEDF alone does not elicit considerable catabolic activity on cartilage under normal conditions. However, its role is more prominent when chondrocytes are challenged with an inflammatory cytokine, suggesting that PEDF enhances the competence to respond to pro-catabolic factors. This concept of competence in the musculoskeletal system has been observed in normal biology. For example, Parathyroid Hormone-related Protein (PTHrP) can provide competence for a subset of chondrocytes to respond to Indian hedgehog (Ihh) signaling, promoting proliferation [61]. Also, other prochondrogenic signals render chondrocytes competent to activate hypertrophic gene expression in response to Runt-related transcription factor 2 (Runx2) [62]. The concept of competence has also been observed in disease states. For instance, glucocorticoids or acute stress sensitize hippocampal microglia to a potentiated pro-inflammatory cytokine response to inflammatory stimuli [63]. Complement factors induced by traumatic lung injury have also been implicated in priming cells toward enhanced inflammatory responses upon immunologic challenge [64]. In the context of arthritis, however, very little is known about competence toward inflammatory stimuli. We did not observe a difference in IL-1β-induced NF-κB nuclear localization upon PEDF overexpression; however, PEDF is known to bind extracellular matrix (ECM) components [51–53], which may also mediate cytokine activities. Additional studies designed to better examine this phenomenon may help to identify the additional compounding factors important in determining the responsiveness or susceptibility of chondrocytes to pro-catabolic factors.

An additional intriguing element is the age-dependent nature of PEDF loss on cartilage integrity under IL-1β treatment. Specifically, PEDF appears to sensitize the cellular response to pro-inflammatory stimuli in aged animals. This is particularly interesting as aging is one of the most prominent risk factors for developing arthritic diseases, but the exact nature of this phenomenon is still not understood. Several reports indicate that age reduces chondrocyte responsiveness to pro-anabolic factors such as insulin-like growth factor 1 (IGF-I) [65–67], and increases susceptibility to osteoarthritis [54], indicating a complicated interplay between aging and inflammation [68]. Consistent with this notion is our observation that cartilage from older wild type bones (29 weeks old) exhibited greater damage compared to younger wild type bones (10 weeks old) upon IL-1β treatment. In particular, PEDF loss prevented this age-related increase in cartilage damage under pro-inflammatory conditions. Thus, it is plausible that PEDF has primed aged chondrocytes to become more responsive to inflammatory stimuli.

The underlying mechanism for our observations is not known; our studies suggest several potential lines of investigation. PEDF levels are reduced in the skin [69] and retinal pigmented epithelium with age [70], but elevated in the kidney [71]; however, changes in PEDF protein levels in the joint with aging are not known. Future studies should explore the effect of aging on PEDF protein levels in the joint. Many receptors have been identified for PEDF [35, 72–74], but the particular receptor or set of receptors for PEDF in chondrocytes remains elusive. Identifying the specific receptor(s) involved may offer clues to the specific signaling pathways important in eliciting the exacerbated response to inflammatory stimuli and may begin to tease out the mechanisms underlying this observation. RNA arrays that characterize changes in gene expression with osteoarthritis over the course of aging may identify additional approaches.

## Conclusion

Our study is the first to show that PEDF exacerbates cartilage degeneration in an age-dependent manner under an inflammatory setting. This is particularly relevant as age is the greatest risk factor for developing degenerative joint disease. PEDF is known to have diverse functions in different tissues and organs. Our findings continue to highlight the multi-faceted activities of PEDF.

## Additional files

Additional file 1:
**Datasets.** Datasets used for quantification. In figures where data are quantified, the datasets are included in tabs of this Excel file labeled by figure number or data described. The “Fig. 2” tab includes cycle threshold numbers for the qRT-PCR data as well as the calculated concentrations of MMP-1, MMP-3, MMP-13 and PEDF from NHAC transduced with LV-GFP or LV-PEDF shown in Fig. 2. The “Fig. 4” tab shows the values for percent surface area loss as determined from the Toluidine Blue images of metatarsal bones. The “Fig. 5” tab shows the values for percent positive MMP-13 area as determined from the MMP-13 images of metatarsal bones. The “Fig. 6” tab shows the values for the percent area loss as determined from the Safranin O/Fast Green/Hematoxylin images of MIA-injected knees. The “Cell Number” tab shows the values for the cell counts as determined from the DAPI and bright field images of MIA-injected knees as referenced in the manuscript. (XLSX 44 kb)
Additional file 2: Figure S1. Immunofluorescence controls. The supplemental figure includes images of sections stained in parallel with each respective staining protocol which received no primary antibody. (PPTX 2875 kb)

## Abbreviations

DAPI: 4’,6-diamidino-2-phenylindole dihydrochloride
ECM: Extracellular matrix
ELISA: Enzyme-linked immunosorbent assay
GFP: Green fluorescent protein
IGF-I: Insulin-like growth factor 1
IHC: Immunohistochemistry
Ihh: Indian hedgehog
IL-1Ra: Interleukin 1 receptor antagonist
IL-1β: Interleukin 1 beta
LV: Lentiviral
MCP-1: Monocyte chemoattractant protein 1
MIA: Monosodium iodoacetate
MMP: Matrix metalloproteinase
NF-κB: Nuclear factor of kappa light polypeptide gene enhancer in B-cells
NHAC: Normal human articular chondrocyte
OA: Osteoarthritis
OARSI: Osteoarthritis Research Society International
PEDF: Pigment epithelium-derived factor
PFA: Paraformaldehyde
PTHrP: Parathyroid hormone-related protein
RA: Rheumatoid arthritis
Runx2: Runt-related transcription factor 2
TBP: TATA-binding protein
TNFα: Tumor necrosis factor alpha
