# Supplementary material for: Pigment Epithelium-Derived Factor (PEDF) mediates cartilage matrix loss in an age-dependent manner under inflammatory conditions
Source: BMC Musculoskelet Disord. 2017 Jan 25;18:39. doi: 10.1186/s12891-017-1410-y (PMC5264335; doi:10.1186/s12891-017-1410-y)
Supplement: Additional file 2: Figure S1. — Immunofluorescence controls. The supplemental figure includes images of sections stained in parallel with each respective staining protocol which received no primary antibody. (PPTX 2875 kb) [file 12891_2017_1410_MOESM2_ESM.pptx]

## Slide 1
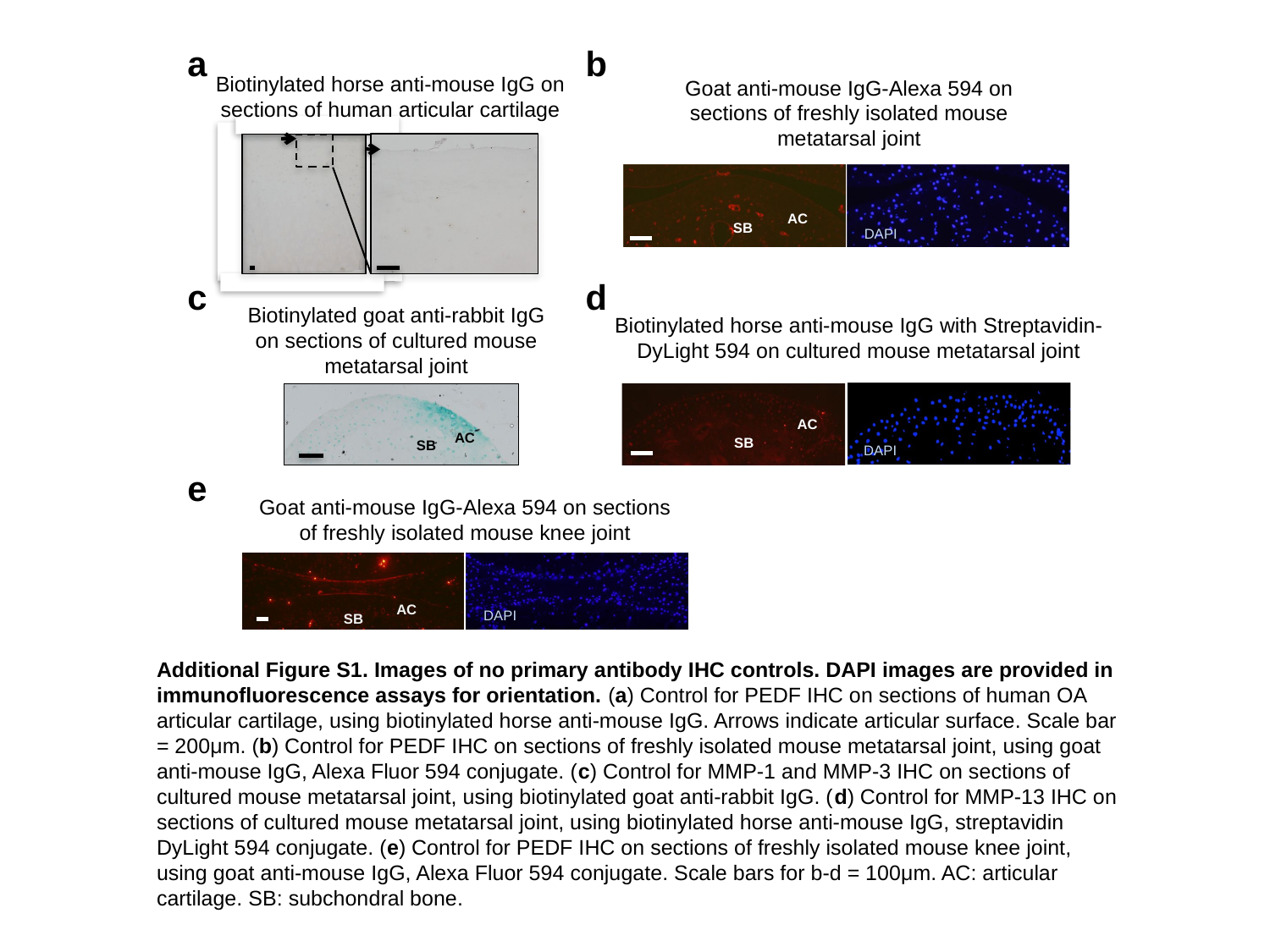

a
b
Biotinylated horse anti-mouse IgG on sections of human articular cartilage
Goat anti-mouse IgG-Alexa 594 on sections of freshly isolated mouse metatarsal joint
AC
SB
DAPI
c
d
Biotinylated goat anti-rabbit IgG on sections of cultured mouse metatarsal joint
Biotinylated horse anti-mouse IgG with Streptavidin-DyLight 594 on cultured mouse metatarsal joint
AC
SB
AC
SB
DAPI
e
Goat anti-mouse IgG-Alexa 594 on sections
of freshly isolated mouse knee joint
AC
DAPI
SB
Additional Figure S1. Images of no primary antibody IHC controls. DAPI images are provided in immunofluorescence assays for orientation. (a) Control for PEDF IHC on sections of human OA articular cartilage, using biotinylated horse anti-mouse IgG. Arrows indicate articular surface. Scale bar = 200μm. (b) Control for PEDF IHC on sections of freshly isolated mouse metatarsal joint, using goat anti-mouse IgG, Alexa Fluor 594 conjugate. (c) Control for MMP-1 and MMP-3 IHC on sections of cultured mouse metatarsal joint, using biotinylated goat anti-rabbit IgG. (d) Control for MMP-13 IHC on sections of cultured mouse metatarsal joint, using biotinylated horse anti-mouse IgG, streptavidin DyLight 594 conjugate. (e) Control for PEDF IHC on sections of freshly isolated mouse knee joint, using goat anti-mouse IgG, Alexa Fluor 594 conjugate. Scale bars for b-d = 100μm. AC: articular cartilage. SB: subchondral bone.
